# A search for bacteria identified from cerebrospinal fluid shunt infections in previous surgical events

**DOI:** 10.1371/journal.pone.0311605

**Published:** 2024-10-10

**Authors:** Paul Hodor, Christopher E. Pope, Kathryn B. Whitlock, Patrick J. McDonald, Jason Hauptman, Lucas R. Hoffman, David D. Limbrick, Tamara D. Simon, on behalf of the Cerebrospinal FLuId MicroBiota in Shunts (CLIMB) Study Group

**Affiliations:** 1 Aurynia LLC, Seattle, Washington, United States of America; 2 Department of Pediatrics, University of Washington, Seattle, Washington, United States of America; 3 New Harmony Statistical Consulting LLC, Clinton, Washington, United States of America; 4 Section of Neurosurgery, University of Manitoba, Winnipeg, Manitoba, Canada; 5 Winnipeg Children’s Hospital, Winnipeg, Manitoba, Canada; 6 Seattle Children’s Research Institute, Seattle, Washington, United States of America; 7 Seattle Children’s Hospital, Seattle, Washington, United States of America; 8 Department of Neurosurgery, University of Washington, Seattle, Washington, United States of America; 9 Department of Neurosurgery, Washington University in St. Louis, St. Louis, Missouri, United States of America; 10 St. Louis Children’s Hospital, St. Louis, Missouri, United States of America; 11 Department of Pediatrics, University of Southern California, Los Angeles, California, United States of America; 12 The Saban Research Institute, Los Angeles, California, United States of America; 13 Children’s Hospital Los Angeles, Los Angeles, California, United States of America; University of Illinois Urbana-Champaign, UNITED STATES OF AMERICA

## Abstract

Shunt infections are a common complication when treating hydrocephalus by cerebrospinal fluid (CSF) shunt placement. The source of infecting pathogens is not well understood. One hypothesis, which we explored here, is that microorganisms persist chronically in the host long before a symptomatic infection occurs and may be detectable in surgical events preceding infection. A cohort of 13 patients was selected, for which CSF samples were available from an infection episode and from a previous surgery event, which was either an initial shunt placement or a revision. Microbiota were analyzed both directly from CSF and from isolates cultured from CSF on aerobic and anaerobic media. The detection and identification of bacteria was done with high throughput DNA sequencing methods and mass spectrometry. The presence of bacteria was confirmed in 4 infection samples, of which 2 were after initial placement and 2 after revision surgery. Taxonomic identification was consistent with clinical microbiology laboratory results. Bacteria were not detected in any of the CSF samples collected at the time of the previous surgical events. While our findings do not provide direct evidence for long-term persistence of pathogens, they suggest the need for consideration of additional source material, such as biofilm and environmental swabs, and/or the use of more sensitive and specific analytical methods.

## Introduction

Cerebrospinal fluid (CSF) shunt placement has been the established treatment for hydrocephalus for over 60 years [1]. CSF shunts allow children with congenital hydrocephalus to survive infancy and allow children with acquired hydrocephalus to avoid further brain injury. Despite their benefits, CSF shunts can cause new and chronic surgical and medical problems. Mechanical malfunction and infections are frequent, leading 60% of shunts to require surgical revision within 4 years [2–4].

CSF shunt infections are a particularly challenging clinical problem. Treatment requires both surgical and medical management. Surgical treatment includes a minimum of two surgeries to remove and replace the infected CSF shunt [5–11], usually including shunt removal and insertion of an interim external ventricular drain [8, 12–16]. Intravenous antibiotic (or antifungal) treatment is usually tailored to the susceptibilities of the organism recovered in conventional microbiological culture. The length of antibiotic treatment is typically 10 to 14 days and is often based on the duration of time that cultures are positive. CSF shunt replacement generally does not occur until the CSF is culture-negative. Despite this aggressive treatment, re-infection rates range from 20 to 25% [7–9].

Currently, diagnosis of CSF shunt infection relies on the recovery of a culturable pathogen from CSF in the clinical microbiology laboratory [6, 17]. The most common organisms recovered are *Staphylococcus epidermidis* and *Staphylococcus aureus* [7–9], followed by other bacteria [5, 6, 18, 19] and fungi [20, 21]. Increasing evidence points to a role for a polymicrobial microbiota in CSF shunt infection. Using molecular, culture-independent methods, we have previously identified small amounts of DNA from a variety of bacteria and fungi present in the CSF at the time of infection diagnosis [22, 23].

It has been hypothesized that CSF shunt infections have a chronic and/or recurrent nature. While infections occur after about 6–8% of CSF shunt surgeries [17, 24], re-infection rates range from 20 to 25% [7–9]. When a patient undergoes multiple surgeries, with each subsequent surgery, the risk of CSF shunt infection increases [25–27]. It is possible that organisms are introduced onto the shunt apparatus at the time of surgery and persist in the patient host over long periods of time, spanning multiple surgical interventions.

Another concern is the likely role in shunt infections of bacterial biofilms formed on the surface of the device and tubing [28]. The presence on such surfaces of clusters of cells of multiple bacterial genera, held together by an extracellular matrix, has been shown by both scanning electron [29, 30] and fluorescence microscopy [31]. Biofilms may contribute to the chronic nature of shunt infections, due to their increased resistance to antibiotic treatment and clearance by the immune system.

Extensive investigations have found few associations between clinical factors and shunt infection outcomes. The only patient factors consistently associated with development of first CSF shunt infection include young age and intervening shunt revision surgeries. Risk of first infection is strongly associated with the number of shunt revisions [26, 27, 32, 33]. Re-infection risk is most strongly associated with difficult to treat first infections—including intermittent negative CSF cultures and complications following first infection [9, 34, 35].

Taken together, these findings suggest a need for a more careful characterization of the dynamics of the microbiota over the course of surgical intervention, infection, and treatment. The aim of this study was to determine the progression of CSF shunt microbiota and its association with CSF shunt infection in successive surgical interventions. We examined CSF obtained from children undergoing CSF shunt surgery (either initial placement or revision), who were subsequently diagnosed with CSF shunt infection. We expected to detect diverse microbial DNA in CSF samples collected at both previous surgery and infection, and we hypothesized that there would be significant similarity between the microorganisms detected in samples at both timepoints.

## Methods

### Outline

**Fig 1:**
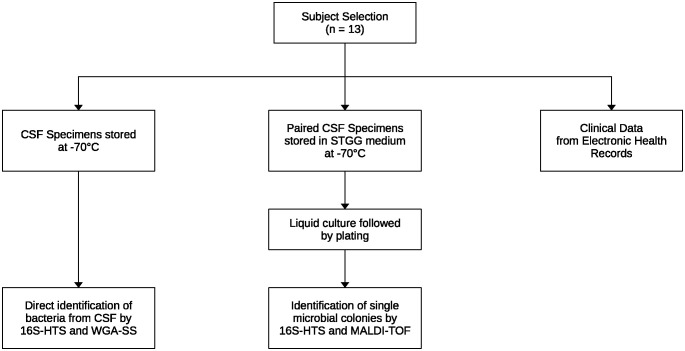
Methods approach. CSF in 2 forms and clinical data were collected from study subjects. CSF and CSF-derived cultures were used for taxonomic identification using multiple experimental approaches.

A diagram of our approach is shown in Fig 1. Each step is described in detail in the following sections. Briefly, from selected patients, CSF was collected either untreated or with the addition of a rich culture medium, and clinical data was extracted from their electronic health records. Taxonomic identification of microorganisms was done directly from CSF samples and from bacterial cultures, using 3 alternative approaches: 16S rRNA high-throughput sequencing (16S-HTS), whole genome amplification followed by shotgun sequencing (WGA-SS), and mass spectrometry.

### Study subjects

Subjects considered for this study were selected from a larger cohort of children under the age of 18, who underwent surgical interventions for treatment of hydrocephalus and were enrolled between August 1st 2014 and January 31st 2022 [36, 37]. Participating sites were Seattle Children’s Hospital, Seattle, USA, BC Children’s Hospital, Vancouver, Canada, and St. Louis Children’s Hospital, St. Louis, USA. Institutional Review Board (IRB) approval was obtained as follows: BC Children’s Hospital (IRB Approval Number: H16–00655), Seattle Children’s Hospital (IRB Approval Number: FWA00002443/PIROSTUDY13446), and St. Louis Children’s Hospital (IRB Approval Number: 201606086). Approval was also secured from the Children’s Hospital of Los Angeles (CHLA), with the IRB approval number CHLA-20–00069. In addition, written informed consent was acquired from the parents or legal guardians of all children in the cohort.

Specific inclusion criteria for this study were that children had to have undergone a CSF shunt initial placement or revision and were subsequently diagnosed with CSF shunt infection. CSF shunt revision is defined as partial or complete surgical intervention on a CSF shunt system. CSF shunt infection is defined as identification of organisms on microbiological culture of CSF fluid obtained from a CSF shunt system.

### Specimen collection

From children who underwent initial CSF shunt placement, CSF samples were obtained from free flowing CSF as well as through the newly placed device in the operating room at the time of surgery. For children undergoing CSF shunt revision, CSF samples were obtained either from a CSF shunt tap or in the operating room at the time of the CSF shunt revision.

CSF samples from children with CSF shunt infection were obtained initially either from a CSF shunt tap or in the operating room at the time of the first CSF shunt surgery. Subsequent CSF samples were generally obtained via external ventricular drain.

All sample collection was done using sterile technique. Samples were stored at 4°C until aliquoted into vials of ∼100 μl. To some aliquots an equal volume of medium containing skim milk, tryptone, glucose, and glycerol (STGG) [38] was added. Vials were then stored at -70°C.

### Clinical data

At each participating center clinical data for each child was extracted from their electronic health record. Collected data included: (1) details of surgical treatment such as dates and times, approaches taken, use of neuroendoscope, use of ultrasound, case duration, shunt type, antibiotic impregnated catheter tubing use; (2) microbiology lab results from culturing CSF using routine aerobic techniques in hospital-certified laboratories, which included dates, organisms recovered, and timing of growth.

### Culture of CSF samples stored in STGG

STGG medium was developed as an additive for optimal recovery of microorganisms from frozen biological specimens [38]. Storage of CSF samples in STGG medium and culturing in rich media in aerobic and anaerobic conditions is more sensitive at detecting the presence of bacteria than high-throughput sequencing methods and may detect species missed by standard clinical laboratory methods [37]. CSF samples stored in STGG medium were cultured to recover any existing bacteria as previously described [37]. Each sample was cultured in VersaTREK REDOX EZ Draw bottles (Thermo Fisher Scientific, MA, USA) of REDOX 1 aerobic and REDOX 2 anaerobic media. Positive controls consisted of laboratory strains of *Escherichia coli* for aerobic and *Cutibacterium acnes* for anaerobic cultures. Uninoculated bottles were used as negative controls. After 10 days, liquid cultures were plated onto blood agar and incubated in aerobic or anaerobic conditions respectively. Any growth that developed on an agar plate was considered evidence for the presence of bacteria in the original CSF sample. Colonies from such plates were used for downstream identification of the organisms.

### 16S rRNA analysis

DNA was extracted from CSF samples, quantitated by qPCR, and analyzed by 16S-HTS as described [36]. In addition, DNA was extracted from cultures of single colonies obtained from STGG-stored CSF samples (above). Negative controls included “extraction” controls, in which CSF was omitted, and “no-template” controls, where PCR was performed without addition of sample DNA. Positive controls were serial dilutions of a mock community [36] and laboratory cultures of *Cutibacterium acnes* and *Staphylococcus aureus*. The 16S rRNA gene amplicon libraries were prepared for the Illumina MiSeq system (Illumina, CA, USA), following the manufacturer’s protocol. The forward primer (5’-TCGTCGGCAGCGTCAGATGTGTATAAGAGACAGCCTACGGGNGGCWGCAG-3’) and reverse primer (5’-GTCTCGTGGGCTCGGAGATGTGTATAAGAGACAGGACTACHVGGGTATCTAATCC-3’) consisted of the 16S rRNA V3-V4 targeting sequences, an Illumina adapter, and a 5’ overhang. Samples were amplified in triplicate and dual-indexed. Libraries were quantified, pooled, and denatured. After sequencing on the MiSeq platform, counts of amplicon sequences were corrected using DADA2 v. 1.6.0 as described [39], and sequences were assigned to taxonomic categories by alignment to the SILVA 16S v. 132 reference database [40].

### Whole genome amplification and shotgun sequencing

WGA-SS has previously proven the capacity to provide strain-specific identification of bacteria and was performed essentially as described [41]. Whole genome DNA was purified from CSF samples and amplified using random primers with the REPLI-g Mini Kit (Qiagen, MD, USA) following the manufacturer’s instructions. Library preparation and sequencing was done on the HiSeq 2500 platform (Illumina, CA, USA), producing paired-end reads of 150 base pairs.

Taxonomic assignment of individual reads was done with Kraken [42], using a custom database, which consisted of complete genomic sequences of human, fungi, bacteria, archaea, and viruses. Estimation of taxon frequencies was done with Bracken [43], using Kraken results as input.

More precise taxonomic assignment was achieved with an assembly approach. Human reads, as assigned by Kraken, were removed from the sequencing data. The remaining reads were assembled *de novo* into scaffolds with SPAdes [44] and metaSPAdes [45]. All resulting scaffolds longer than 1000 base pairs were aligned with nucleotide BLAST [46] against the non-redundant NCBI nucleotide collection (https://www.ncbi.nlm.nih.gov/nucleotide) to identify the closest matching organisms. Matches were compared between surgery and infection episodes of the same patient and with the microbiology lab results.

### Mass spectrometry for classification of bacteria

A mass spectrometry method was applied for taxonomic identification of cultured bacteria as described [37]. Bacteria were classified using matrix-assisted laser desorption/ionization—time of flight (MALDI-TOF) mass spectrometry and analyzed on a Microflex LT Biotyper instrument (Bruker Daltonics, Leipzig, Germany). In brief, individual colonies were picked from an agar plate (see above) and homogenized with a matrix solution. The mixture was then applied to a MALDI target plate and allowed to dry. The plate was inserted into the MALDI-TOF instrument for analysis. The resulting mass spectrum was compared to a database of known bacterial spectra to identify the closest match to the unknown bacterial sample being tested.

## Results

### Patients and CSF samples

Patients in this study had a surgical procedure either for initial CSF shunt placement or CSF shunt revision and subsequently developed an infection. CSF samples were collected both from the time of previous surgery and from the infection episode. Microbiology lab culture results were available for all infections.

There were 13 patients who met these criteria between 2016 and 2019, distributed between two sites. The time interval between CSF samples collected at previous surgery and infection ranged from 1 to 211 days, with a mean of 39 days and a median of 24 days. For presentation purposes, a subset of 8 patients was selected, who all had microbiology culture done at previous surgery and had a single microorganism recovered in culture at the infection time point (Table 1). The reduced data set is sufficient to support the findings and conclusions of this work. The full list of patients is available as supplementary information in S1 Table.

**Table 1:** Subset of patients whose CSF samples were analyzed in this study. A first CSF sample was collected during the previous surgery and a second during the subsequent infection episode. Column “Days” shows the time interval between previous surgery and infection samples. Results from standard clinical microbiology culture are shown. Exceptions are indicated when the date of the culture sample differed from the date of the sample used in this study. 5 patients are not shown in this table, who were not tested by microbiology culture at the previous surgery and/or had polymicrobial results during the infection episode. The full list of patients is available in S1 Table.

Patient	Site	Previous surgery	Days	Microbiology culture, previous surgery	Microbiology culture, infection
P01	Site 1	Initial shunt placement	14	negative	*Staphylococcus aureus*
P02	Site 1	Initial shunt placement	27	negative	*Staphylococcus capitis*
P03	Site 2	Shunt revision	45	negative	*Staphylococcus aureus* 1 day prior to the infection sample
P04	Site 2	Shunt revision	1	negative	*Staphylococcus lugdunensis*
P06	Site 2	Shunt revision	3	negative	*Enterobacter cloacae*
P11	Site 1	Shunt revision	11	negative	*Staphylococcus epidermidis* 1 and 2 days prior to the infection sample
P12	Site 1	Shunt revision	54	negative	*Streptococcus mitis*
P13	Site 1	Initial shunt placement	9	negative	*Klebsiella aerogenes*

Microbiological culture identified a diverse array of microorganisms in infection samples (Table 1). In most cases cultures yielded one unique species, with a preponderance of the genera *Staphylococcus* and *Streptococcus*. The one exception was *Staphylococcus aureus*, which was identified in 2 patients, one from each site.

At the time of previous surgery most samples were culture-negative. There were 2 cases in which *Cutibacterium acnes* was identified (S1 Table). One of them was considered likely to represent contamination, as growth was only detected in long-term broth culture.

Of note, the CSF samples which were analyzed by high-throughput sequencing methods in this study were not always the same as those used in standard microbiological culture. There were several instances when there was at least a day difference from when a positive culture result was reported (Table 1).

### Taxonomic analysis of CSF samples

**Fig 2:**
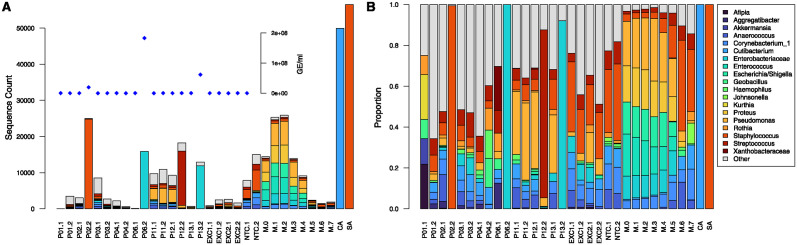
Taxonomic distribution of 16S RNA amplicons shown as raw counts (A) and proportions (B). Patient labels are as in Table 1, with the number after the dot indicating previous surgery (1) or infection (2). Negative controls consist of extraction (EXC) and no-template (NTC) controls. Positive controls are the mock community (M.n), where n is the 10^n^ dilution factor, and pure cultures of *Cutibacterium acnes* (CA) and *Staphylococcus aureus* (SA). Taxonomic categories that appeared in at least one sample at a proportion of 0.1 or more are given by name. The inset shows qPCR measurements of 16S RNA in genome equivalents (GE) per ml. Data for all 13 patients is shown in S1 Fig.

The total bacterial load in all patient CSF samples was quantitated by 16S qPCR. The results, plotted as an inset in Fig 2A, show 3 points that stand out with high qPCR values. These are samples from patients P02, P06, and P13, all from the infection episode, which had qPCR values of at least 2 × 10^7^ GE/ml. An additional infection sample, from P12, was qPCR-positive with a value of 3.2 × 10^5^ GE/ml, which is too low to be visibly noticeable on the plot. All other samples were below the limit of detection of 5 × 10^4^ GE/ml.

Taxonomic identification of bacteria present in the CSF samples was done by 16S-HTS (Fig 2). The four infection samples (P02.2, P06.2, P12.2, P13.2) with high and intermediate qPCR values had the highest sequence counts (Fig 2A). Also, they had a single, predominant taxon, which constituted between 82.3 and 99.9% of sequences (Fig 2B). The remaining samples, with low qPCR values, displayed a mixture of taxa, the composition of which was similar to that of extraction and no-template negative controls. They represent common microorganisms and include those originally identified by laboratory culture (Table 1). It is therefore difficult to confidently claim the presence of any particular taxon in the low qPCR samples from the 16S-HTS results.

A summary of the taxonomic assignments of the 4 16S-HTS-positive samples is shown in Table 2. Due to the limitation of this method, identification of the prevalent microorganism was restricted to the family (*Enterobacteriaceae*) or genus level (*Staphylococcus*, *Streptococcus*). These assignments were consistent with the microbiology culture results, which were available at the species level (cf. columns 2 and 3 in Table 2).

**Table 2:** Identification of microorganisms by high throughput methods in CSF and culture. The first 2 columns show the patient id and microorganism identified by clinical microbiology culture (see Table 1 for details). The next 2 columns show the most abundant microorganism by sequence count and its percentage by direct sequencing from CSF. The last 2 columns show the taxonomic identification by 2 different methods of colonies recovered by culturing CSF samples stored in STGG medium.

Patient	Clinical microbiology culture	16S-HTS CSF top species (%)	WGA-SS CSF top species (%)	16S from STGG culture	MALDI-TOF from STGG culture
P02	*Staphylococcus capitis*	*Staphylococcus* (99.4)*	*Staphylococcus capitis* (64.8)	*Staphylococcus*	*Staphylococcus capitis*
P06	*Enterobacter cloacae*	*Enterobacteriaceae* (99.9)*	*Enterobacter hormaechei* (68.9)	*Enterobacter*	*Enterobacter cloacae*
P12	*Streptococcus mitis*	*Streptococcus* (82.3)	*Serratia marcescens* (11.2)**	NA***	NA***
P13	*Klebsiella aerogenes*	*Enterobacteriaceae* (92.1)*	*Klebsiella aerogenes* (23.4)	*Klebsiella*	*Klebsiella aerogenes*

* Samples with qPCR ≥ 2 × 10^7^ GE/ml

** *Streptococcus spp.* absent in this sample

*** Not Available, no growth in culture

**Fig 3:**
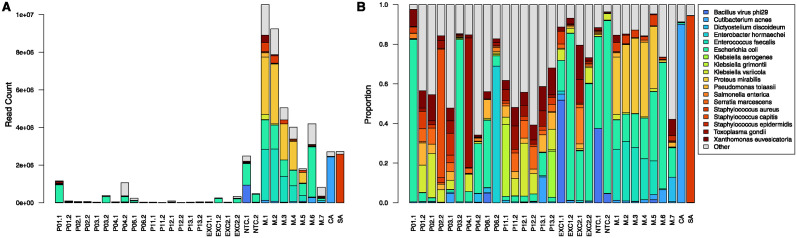
Taxonomic distribution of WGA-SS non-human reads shown as raw counts (A) and proportions (B). Labels for patients and control samples are as in Fig 2. Data for all 13 patients is shown in S2 Fig.

Identification of microorganisms in CSF samples was also done with WGA-SS. Human reads, as determined by Bracken, made up between 43% and 99% of total reads, with a mean of 87% and median of 92%. After removal of human reads, remaining read counts ranged between 2,000 and 1.2 million per sample, with a mean of 210,000 and median of 51,000 (Fig 3A). These values were in the same range as negative controls and generally lower than for positive controls.

Taxonomic profiles of WGA-SS non-human reads (Fig 3A and 3B) were noisier than that those of 16S-HTS, with higher proportions in CSF samples of taxons that were also present in negative controls. There was good concordance between the 2 methods (Table 2) in the sense that the most abundant taxon in the 3 qPCR-positive samples matched the top species identified by 16S-HTS. The one exception was sample P12.2, in which *Streptococcus* was identified by 16S-HTS, but was absent in WGA-SS. Similar to 16S-HTS, the presence of species identified by microbiology culture could not be confirmed by WGA-SS with confidence.

### Search for microorganisms at previous surgery

The main goal of this study was to search for evidence that microorganisms responsible for CSF infection are present at the time of previous surgery. As expected clinically, microbiology lab cultures did not provide such evidence; almost all patients had negative culture results at previous surgery. Positive cultures at previous surgery occurred in 2 patients, but the identified microorganism, *C. acnes*, was not present in the infection sample. There were 3 patients for which microbiology lab results were not available at previous surgery.

Two other lines of evidence were pursued to search for infecting microorganisms at the time of previous surgery. The first relied on the partial genomic sequences assembled from WGA-SS reads. Based on similarity search of assembled WGA-SS reads, we were unable to find any sequences that would indicate the persistence of a microorganism from previous surgery to infection. Considering in particular the 3 qPCR-positive infection samples, the constructed assemblies of the dominant microorganism had low genomic coverage, with a total nucleotide count on the order of 10 to 100 kb. In corresponding previous surgery samples, recovered sequences did not contain matching organisms, not even at the genus level.

The second line of evidence pursued to search for infecting microorganisms at the time of previous surgery involved culturing of CSF samples stored in STGG. When our study samples were cultured both under aerobic and anaerobic conditions, growth occurred only in the 3 infection samples that were also qPCR-positive. Taxonomic identity of colonies was determined by MALDI-TOF MS and was consistent with results from the other experimental methods (Table 2). Lack of growth in samples at the previous surgeries means that this approach yielded no evidence for persistence of microorganisms from surgery to infection.

On a side note, the taxonomic assignments by different methods summarized in Table 2 shows an apparent discrepancy for patient P06. WGA-SS identified the species as *E. hormaechei*, while both clinical lab and MALDI-TOF as *E. cloacae*. Detailed examination of BLAST alignments of contigs obtained from WGA-SS reads showed matches of over 99% identity with database entries for both species, but slightly higher for *E. hormaechei*. Clearly the 2 species are closely related, and conclusive assignment of an isolate to one or the other may require careful consideration of multiple types of data points.

## Discussion

In this study we considered patients, who had undergone a surgical intervention for initial CSF shunt placement or revision, and had a subsequent CSF shunt infection. We searched for the presence of microbiota in CSF from the previous surgery as a potential cause of the later infection. Detection of microorganisms relied on 3 different molecular techniques applied directly to untreated CSF or to cultures of CSF that had been stored in STGG medium. Although we identified several types of bacteria during the infection episode, there was no evidence for the presence of these or any other microorganism in CSF at the previous surgery.

One limitation of the experimental approach is highlighted by the fact that we detected microorganisms in only 4 out of 13 samples from the infection episodes, which had all been positive by clinical microbiology lab culture. For some of the cases, this finding was likely due to the difference in collection time of the available CSF sample, which was one or more days later than the sample used for clinical microbiology lab culture. Antibiotic treatment may have rapidly cleared CSF of bacteria by the time our samples were collected. Another potential reason for negative results is suboptimal conditions during handling and long-term storage of CSF samples. In a previous study [41] we have seen evidence of DNA degradation in our CSF sample collection, which could impact up to 5% of samples. Finally, our current and previous results suggest that even during an active infection, bacterial load of CSF can be very low. Thus, our methods often operate near their limit of detection and may produce false negatives due to insufficient sensitivity.

When infection follows an initial shunt placement, the source of the infecting microorganism could be internal or external. For example, an internal source would involve a microorganism naturally present in the patient’s ventricular system. Although CSF has been hypothesized to be sterile, a search for bacteria using high sensitivity methods could not rule out the presence of bacteria in CSF from children with hydrocephalus [37]. External sources could include the patient’s epidermal flora and the operating room environment.

An experimental approach to address the source of infection after initial shunt placement would need to have enhanced sensitivity compared to methods used so far, while maintaining high specificity. One approach we suggest would be a targeted search using nested PCR with primers developed from the genomic sequence obtained from the infection episode. PCR amplification would provide the needed sensitivity, while selection of strain-specific nested primers would ensure high specificity. Samples to be analyzed would include CSF from initial shunt placement (for the internal source hypothesis) and skin and operating room environmental swabs (for the external source hypothesis).

The number of CSF shunt revisions that a patient has undergone is a major predictor of risk for first shunt infection [25–27]. A possible explanation is that an infection that follows a revision surgery is more likely due to persistence of a microorganism chronically established in the host, compared to an infection that follows initial shunt placement. Therefore, in addition to the analysis of CSF with enhanced methods as above, future studies should also examine the surfaces of shunt catheters, on which adherent biofilms can form. Microorganisms, such as bacteria and fungi, may be more likely to be detected in this surface-attached state on the failed shunt device, where they would be much more concentrated than when suspended in CSF.

These future studies will lead to a better understanding of the source of microorganisms causing infections, as well as their evolution within the host and response to treatment. Such mechanistic understanding will inform improvements in both clinical procedures to reduce the risk of infections as well as antibiotic treatment regimens to more effectively treat infections once they occur.

## Supporting information

S1 Table Complete list of 13 patients, whose CSF samples were analyzed in this study. A first CSF sample was collected during the previous surgery and a second during the subsequent infection episode. Column “Days” shows the time interval between previous surgery and infection samples. Results from standard clinical microbiology culture are shown. Positive results represent microorganisms identified from plate cultures of CSF, unless otherwise noted. Exceptions are indicated when the date of the culture sample differed from the date of the sample used in this study.
(PDF)

S1 Fig Taxonomic distribution of 16S RNA amplicons in all 13 patients shown as raw counts (A) and proportions (B). Labels for patients and control samples are as in Fig 2.
(PDF)

S2 Fig Taxonomic distribution of WGA-SS non-human reads in all 13 patients shown as raw counts (A) and proportions (B). Labels for patients and control samples are as in Fig 2.
(PDF)

S1 File Data used to build plots in Figs 2, 3 and S1 and S2 Figs, including qPCR measurements, counts and proportions of 16 RNA amplicons, and WGA-SS non-human reads. (XLSX)
