# Supplementary material for: A search for bacteria identified from cerebrospinal fluid shunt infections in previous surgical events
Source: PLoS One. 2024 Oct 10;19(10):e0311605. doi: 10.1371/journal.pone.0311605 (PMC11469614; doi:10.1371/journal.pone.0311605)
Supplement: S1 Table — A first CSF sample was collected during the previous surgery and a second during the subsequent infection episode. Column “Days” shows the time interval between previous surgery and infection samples. Results from standard clinical microbiology culture are shown. Positive results represent microorganisms identified from plate cultures of CSF, unless otherwise noted. Exceptions are indicated when the date of the culture sample differed from the date of the sample used in this study. (PDF) [file pone.0311605.s001.pdf]

**S1 Table. Complete list of 13 patients, whose CSF samples were analyzed in this study.** A first CSF sample was collected during the previous surgery and a second during the subsequent infection episode. Column "Days" shows the time interval between previous surgery and infection samples. Results from standard clinical microbiology culture are shown. Positive results represent microorganisms identified from plate cultures of CSF, unless otherwise noted. Exceptions are indicated when the date of the culture sample differed from the date of the sample used in this study.

| Patient | Site | Previous surgery | Days | Microbiology culture, previous surgery | Microbiology culture, infection |
|---|---|---|---|---|---|
| P01 | Site 1 | Initial shunt placement | 14 | negative | *Staphylococcus aureus* |
| P02 | Site 1 | Initial shunt placement | 27 | negative | *Staphylococcus capitis* |
| P03 | Site 2 | Shunt revision | 45 | negative | *Staphylococcus aureus*, culture sample collected 1 day prior to the infection sample |
| P04 | Site 2 | Shunt revision | 1 | negative | *Staphylococcus lugdunensis* |
| P05 | Site 2 | Shunt revision | 24 | *Cutibacterium acnes* from broth only | negative on day of infection sample; *viridans* streptococci from broth only, culture sample collected 10 days after the infection sample; *Staphylococcus epidermidis*, culture sample collected 14 days after the infection sample |
| P06 | Site 2 | Shunt revision | 3 | negative | *Enterobacter cloacae* |
| P07 | Site 2 | Shunt revision | 211 | not tested | *Pseudomonas aeruginosa*, *Streptococcus agalactiae* (Group B) on the day of the infection sample; *Cutibacterium acnes*, culture sample collected 1 day prior to the infection sample |
| P08 | Site 2 | Shunt revision | 24 | *Cutibacterium acnes* | catheter tip *Staphylococcus hominis* and CSF negative on the day of the infection sample; CSF *Bacillus* species, not *B. anthracis*, culture sample collected 1 day after the infection sample |
| P09 | Site 2 | Shunt revision | 59 | not tested | shunt tip/hardware *Candida parapsilosis*, CSF sample negative |
| P10 | Site 2 | Endo 3rd ventriculostomy | 20 | not tested | *Enterococcus* |
| P11 | Site 1 | Shunt revision | 11 | negative | *Staphylococcus epidermidis*, culture samples collected 1 and 2 days prior to the infection sample |
| P12 | Site 1 | Shunt revision | 54 | negative | *Streptococcus mitis* |
| P13 | Site 1 | Initial shunt placement | 9 | negative | *Klebsiella aerogenes* |
