# Supplementary figures and images for: A search for bacteria identified from cerebrospinal fluid shunt infections in previous surgical events
Source: PLoS One. 2024 Oct 10;19(10):e0311605. doi: 10.1371/journal.pone.0311605 (PMC11469614; doi:10.1371/journal.pone.0311605)

**A**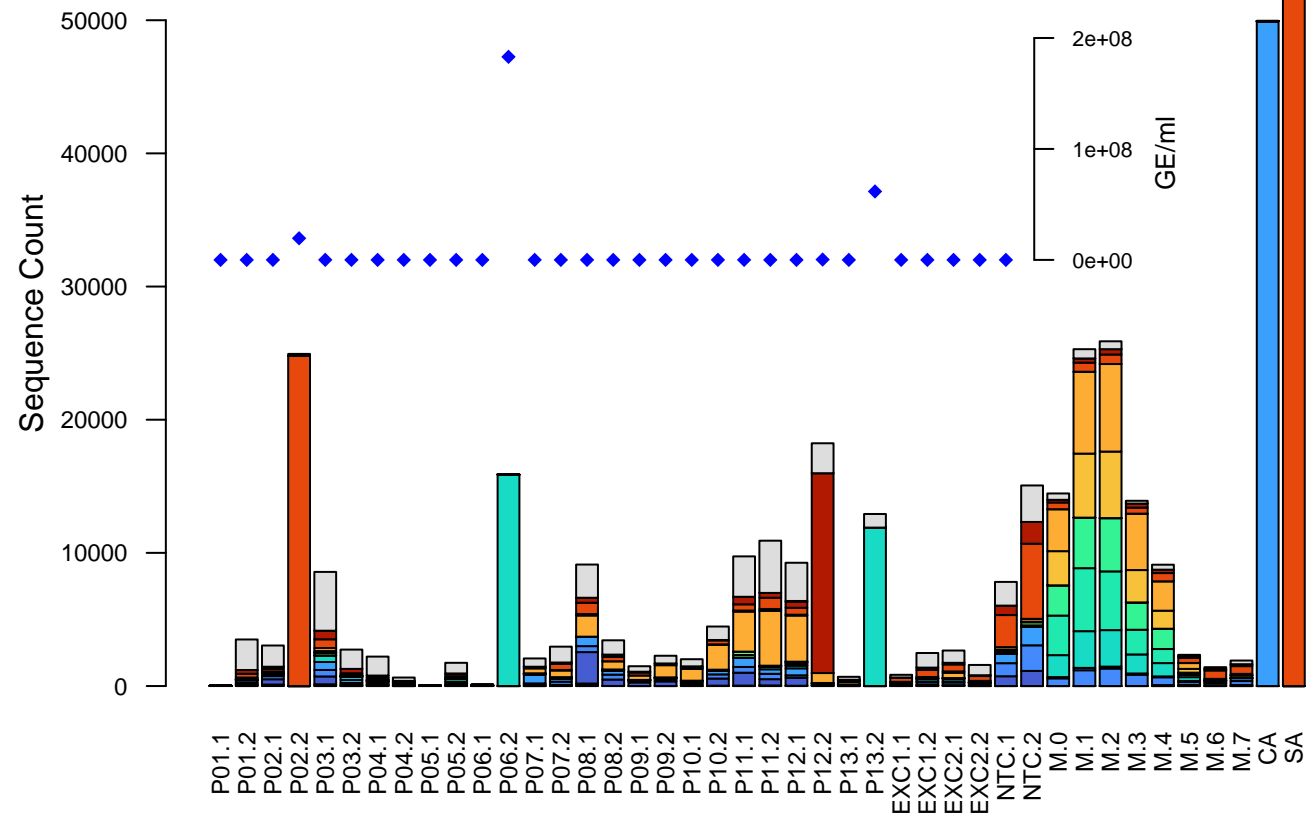**B**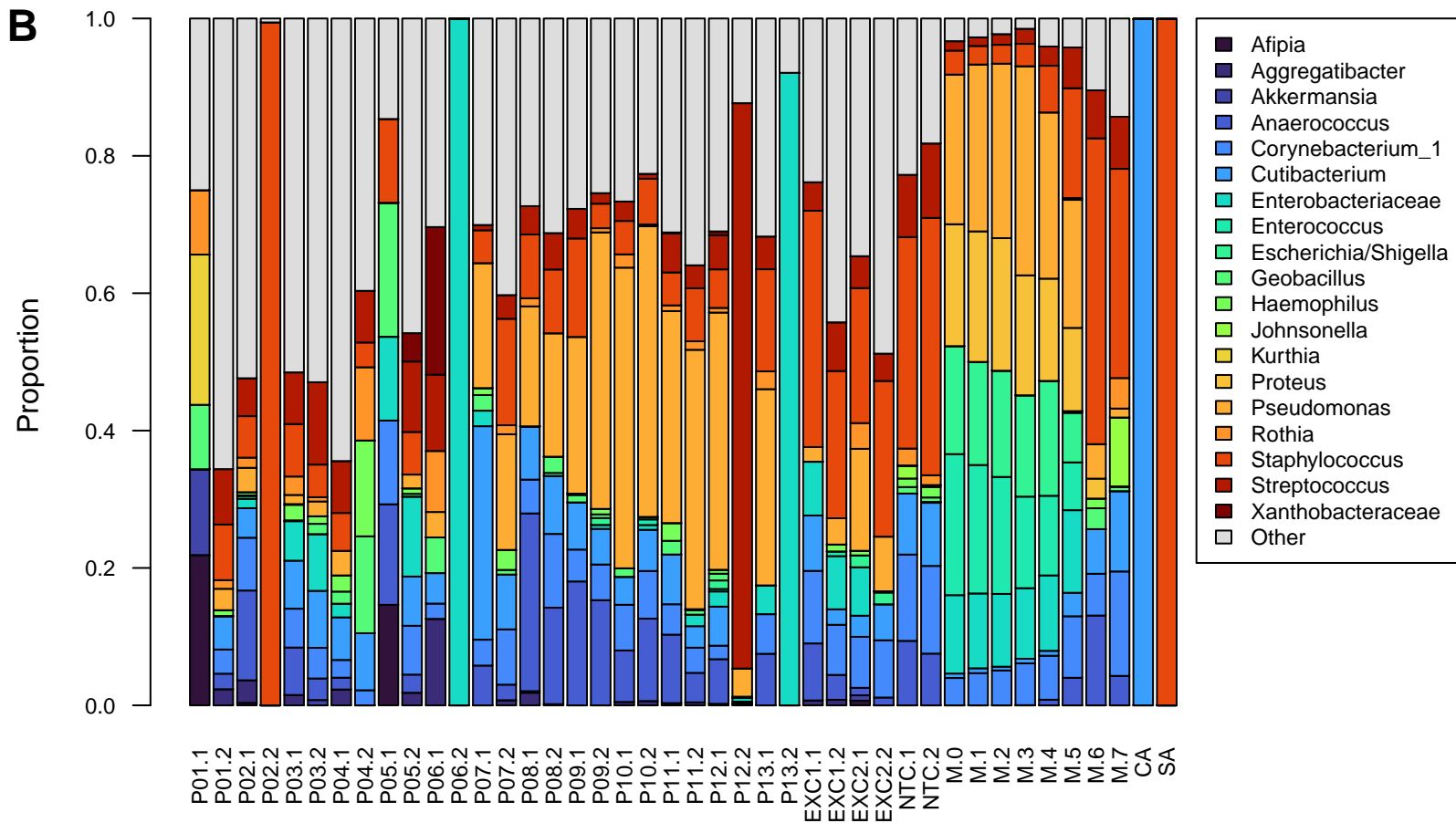

Supplement: S1 Fig — Labels for patients and control samples are as in Fig 2. (PDF) [file pone.0311605.s002.pdf]

**A**

Read Count

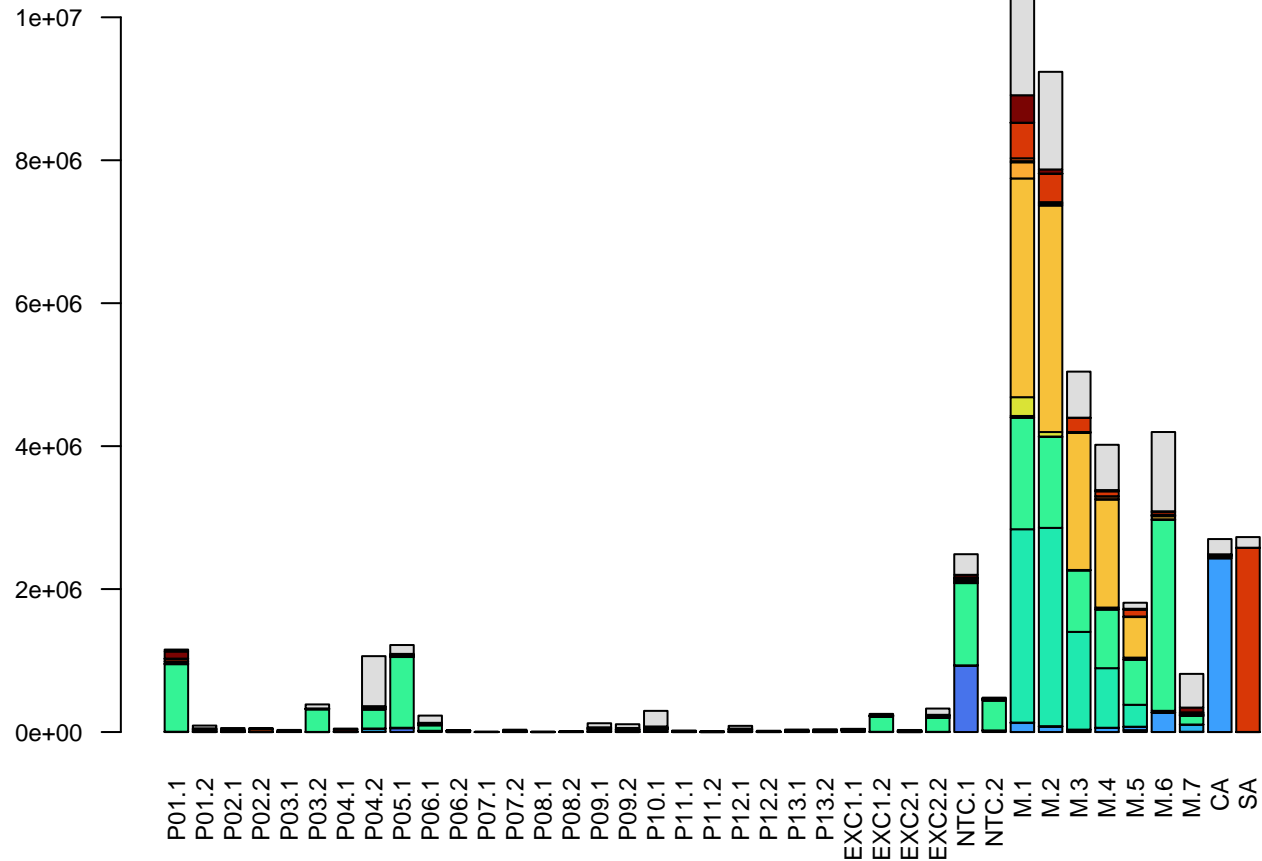**B**

Proportion

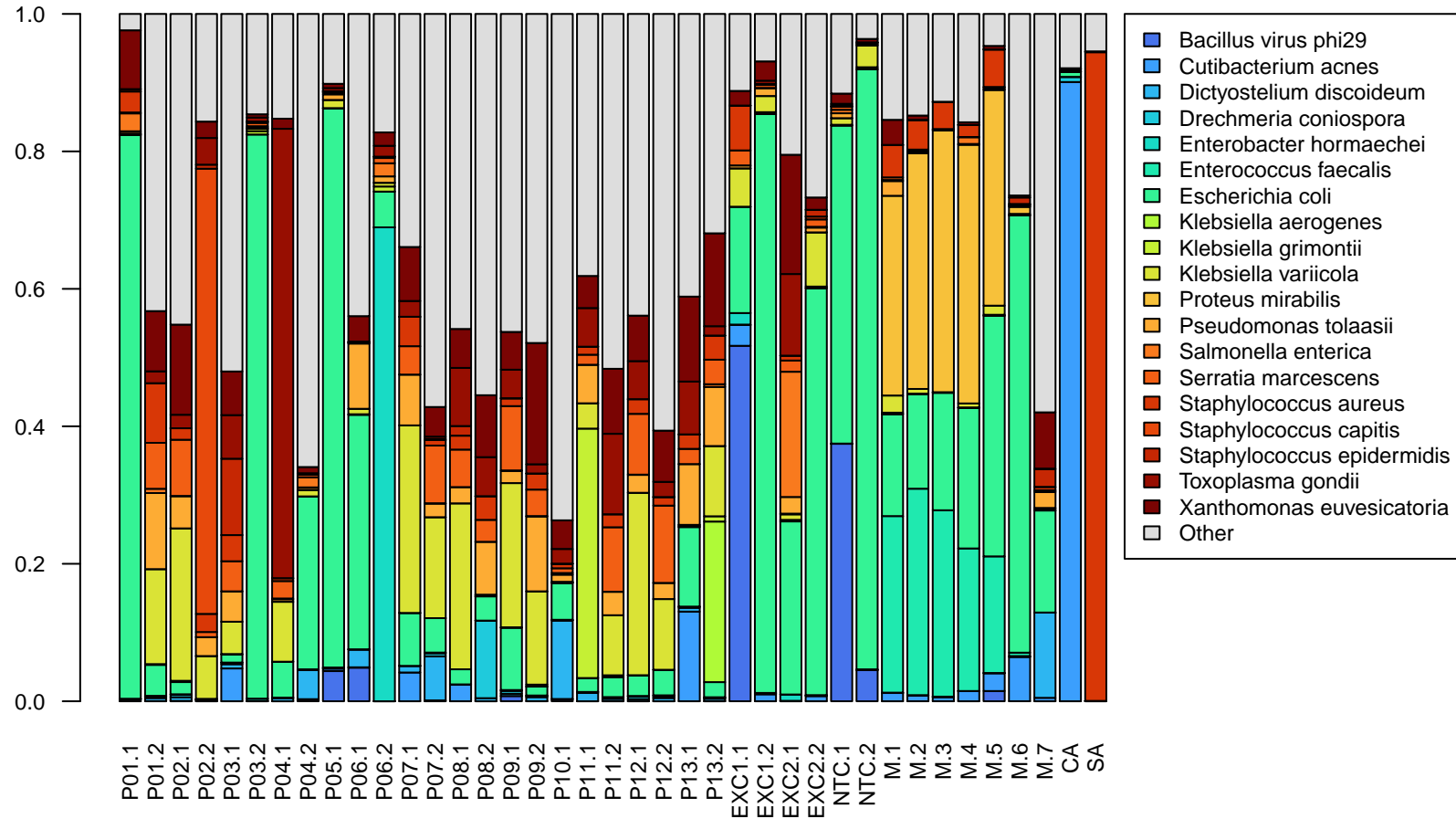

Supplement: S2 Fig — Labels for patients and control samples are as in Fig 2. (PDF) [file pone.0311605.s003.pdf]
